# Elbow Microinstability: From the State of the Art to an Integrated Clinical Approach

**DOI:** 10.3390/jcm14217584

**Published:** 2025-10-25

**Authors:** Nikolaos Platon Sachinis, Valeria Vismara, Pietro Simone Randelli, Paolo Arrigoni

**Affiliations:** 1 First Orthopaedic Department, “Georgios Papanikolaou” Hospital, 57010 Thessaloniki, Greece; nick.sachinis@gmail.com; 2 Scuola di Specializzazione in Ortopedia e Traumatologia, Università Degli Studi di Milano, Via Festa del Perdono 7, 20122 Milan, Italy; vale.vismara@gmail.com; 3 Laboratory of Applied Biomechanics, Department of Biomedical Sciences for Health, Università Degli Studi Di Milano, Via Mangiagalli 31, 20133 Milan, Italy; pietro.randelli@unimi.it; 4 U.O.C. 1° Clinica Ortopedica, ASST Centro Specialistico Ortopedico Traumatologico Gaetano Pini-CTO, Piazza Cardinal Ferrari 1, 20122 Milan, Italy; 5 Research Center for Adult and Pediatric Rheumatic Diseases (RECAP-RD), Department of Biomedical Sciences for Health, Università Degli Studi Di Milano, Via Mangiagalli 31, 20133 Milan, Italy; 6 Clinica Ortopedica, Azienda Socio Sanitaria Territoriale Centro Specialistico Ortopedico Traumatologico Gaetano Pini-CTO, Piazza Cardinal Ferrari 1, 20122 Milan, Italy

**Keywords:** elbow microinstability, Symptomatic Minor Instability of the Lateral Elbow (SMILE), radial collateral ligament complex, lateral elbow pain, arthroscopic stabilization techniques, minor elbow instability, radiocapitellar instability

## Abstract

Lateral elbow pain is a common condition often misattributed solely to tendinopathy, while subtle instability may represent a significant underlying cause. Traditional classifications of elbow instability primarily address traumatic or grossly unstable patterns, leaving minor forms underrecognized. Recent evidence has emphasized the role of the Radial-Lateral Collateral Ligament (R-LCL) in maintaining joint stability, and its elongation has been linked to Symptomatic Minor Instability of the Lateral Elbow (SMILE). This model describes a horizontal type of radiocapitellar instability, where ligamentous incompetence leads to compensatory overload of the extensor carpi radialis brevis, ultimately producing chronic pain. Advances in diagnostic tools—including dynamic ultrasound (HELP-US test), CT arthrography with the SMILE Index, and arthroscopic signs such as the Loose Collar Sign—have improved recognition of this condition. However, surgical controversies remain, particularly regarding the potential destabilizing role of lateral release in patients with unrecognized R-LCL pathology. Arthroscopic stabilization techniques, such as R-LCL plication or imbrication, have shown promising outcomes, offering pain relief and functional recovery with minimally invasive approaches. This review integrates anatomical, biomechanical, and clinical evidence into a structured diagnostic and therapeutic algorithm, aiming to reduce diagnostic uncertainty and guide tailored interventions. Recognition of microinstability, and, in particular, the SMILE model, is crucial to optimize management of patients with chronic lateral elbow pain refractory to conservative measures.

## 1. Introduction

Lateral elbow pain is a frequent clinical condition, often attributed to repetitive overload conditions, which, over time, damage tendon insertions. Lateral epicondylitis affects approximately 1–4% of adults each year, with higher rates observed in individuals engaged in repetitive wrist extension or gripping activities [1,2]. The term has been traditionally attributed to overuse of the extensor muscles, particularly the Extensor Carpi Radialis Brevis (ECRB), leading to inflammation or irritation of the insertion of the tendon on the lateral humeral epicondyle [3]. Despite this, the differential diagnosis for this common condition includes nerve entrapment syndromes, cervical radiculopathy, bony pathology, inflammatory disorders, and subtle forms of elbow instability [4].

While elbow instability often refers to true dislocation or subluxation, it is currently acknowledged that it is not a single condition but rather covers a spectrum of many different forms, from acute to chronic [5]. Posterolateral Rotatory Instability (PLRI) is by far the most commonly described traumatic form of elbow instability, following a tear of the lateral ulnar collateral ligament, allowing external rotation of the ulna on the humerus [6]. Five parameters, timing, articulations, direction, degree, and associated fractures, have been considered for classifying PLRI [7]. On the other side of the spectrum, atraumatic PLRI has been described as a subtle form of elbow instability, mainly characterized by chronic lateral elbow pain that occurs in patients with a positive history for tennis elbow, especially following corticosteroid injection [8,9].

Despite a growing interest in elbow pathology, physicians still lack a validated classification system that could help recognize the subtle differences among elbow joint laxity or subclinical instability. This does not hold for other anatomical districts, such as the shoulder or the wrist. Indeed, the Stanmore classification system [10] is one of the most comprehensive classification systems related to shoulder instability, together with the recently described “AB classification” [11]. In the wrist, the CID-CIND-CIC framework has been widely adopted for carpal instability [12,13,14].

**Figure 1:**
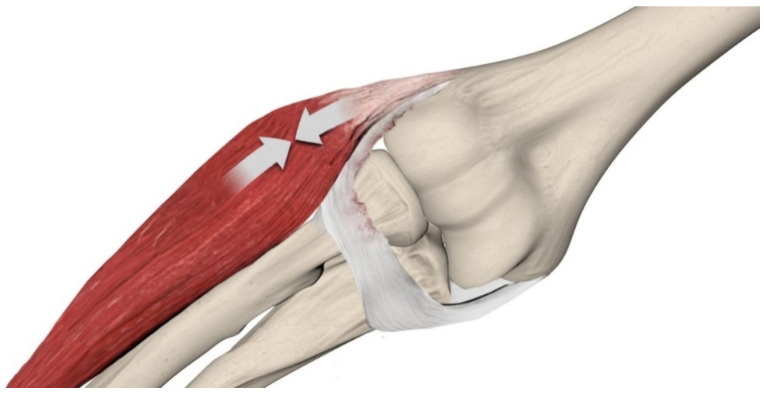
Dynamic stabilization as a consequence of the R-LCL elongation to compensate for a ligamentous incompetence.

This absence of classification in the elbow contributes to ambiguity in diagnosing, reporting, and managing subtle elbow instability that does not classify as PLRI. A shared lexicon and reproducible clinical or biomechanical criteria for minor elbow instability are lacking, which interferes with standardization in diagnosis and treatment pathways. The Symptomatic Minor Instability of the Lateral Elbow (SMILE) model has received increasing attention: introduced in 2017, the authors reported that the majority of patients with atraumatic lateral elbow pain showed intra-articular abnormalities [15] and that the elongation of the Radial-Lateral Collateral Ligament (R-LCL) is involved in the development of horizontal instability of the proximal radioulnar and radio-capitellar joint. According to this condition, ECRB tendinopathy is the dynamic consequence of R-LCL elongation with an attempt to compensate for ligamentous incompetence (Figure 1). During daily desk activities, the elbow is continuously loaded in varus by the weight of the forearm and hand, with a variable flexion between 40 and 90 degrees of flexion and the forearm from neutral to full pronation.

A Later development of the SMILE condition was the SMILE Index and its scoring system, which was introduced by a semiquantitative CT (Computed Tomography) arthrography study [16]. Biomechanical cadaveric investigations confirmed that isolated R-LCL sectioning, while preserving the common extensor origin, led to increased lateral gapping under varus load—suggesting that the R-LCL has a primary role in subtle elbow stability [17]. A recent ultrasonographic (US) study demonstrated that US examination in a weighted varus-flexion position could identify dynamic radio-capitellar widening in symptomatic patients [18].

A non-systematic search of the literature was performed in PubMed and Scopus using the keywords “elbow instability,” “microinstability,” “SMILE,” “LCL,” and “arthroscopy”.

This work explores the concept of elbow micro-instability with an emphasis on SMILE and horizontal radio-capitellar instability, synthesizing evidence from anatomical, biomechanical, imaging, and clinical studies to clarify diagnostic and therapeutic strategies for this under-recognized condition.

## 2. Classification and Terminology

Traditional classification systems of elbow instability have focused on traumatic and grossly unstable conditions. Modern literature, involving the last 30 years, arguably started with Morrey’s classification, where elbow instabilities are defined as acute and chronic [19]. Acute dislocations are further diversified according to severity (complete/incomplete), direction of instability, and accompanying injuries. Chronic instabilities were subclassified as unreduced and recurrent. Ring and Jupiter, two years later, further classified the post-traumatic instabilities in acute and chronic forms [20], and in 2000, O’Driscoll identified timing, articulations involved, direction, degree, and associated injuries as important parameters to elbow instability [7]. Posterolateral rotatory instability (PLRI), valgus instability from MUCL (Medial Ulnar Collateral Ligament) tears, and complex fracture-dislocations are currently well-documented entities with established surgical pathways [6,7,21,22]. More recent proposals like the classification from the Italian Shoulder and Elbow Society, in order to unify the classification, incorporate more features such as onset (acute vs. chronic), soft tissue/soft tissue and bone, and then subcategorize according to complexity, etiology, directionality, and severity [5].

Apart from these frameworks, an increasing number of patients who complain of insidious symptoms of lateral elbow pain present with negative findings on static radiographs and even on Magnetic Resonance Imaging (MRI). The elbow on such occasions may exhibit patterns of load-dependent instability that are best identifiable by using stress imaging techniques or intraoperative arthroscopic assessment. The SMILE model has been introduced to describe atraumatic lateral elbow instability, which may present without radiographic dislocation or positive pivot shift signs.

A cohort analysis showed that 48.6% of patients with unexplained lateral elbow pain and no radiographic dislocation or positive PLRI signs, undergoing diagnostic arthroscopy, displayed signs of R-LCL patholaxity [15]. Similarly, in a radiographic study using the SMILE Index, subtle changes such as elongation of the R-LCL and chondral wear could be demonstrated, providing more evidence on intra-articular findings that may relate to the patient’s pain [16]. As a non-traumatic, functional instability, SMILE likely fits within the “chronic simple” classification but merits distinct recognition. Unlike PLRI, which gives rise to a posterolateral subluxation in the vertical axis of the joint, SMILE presents with a horizontal type of instability of the radial head compared to the lesser sigmoid notch of the ulna, without frank dislocation.

## 3. Anatomical and Biomechanical Considerations

The lateral elbow stabilizing structures consist of a complex network that includes the Lateral Collateral Ligament Complex (LCLC), comprising the Radial Collateral Ligament (RCL), Lateral Ulnar Collateral Ligament (LUCL), annular ligament, and accessory collateral ligaments—as well as the joint capsule and dynamic stabilizers like the extensor tendons. The integrity and interaction of these elements are crucial for maintaining elbow stability under varus and rotational loads.

The radial band of the lateral collateral ligament complex and the ECRB play a synergistic role in maintaining lateral elbow stability. The ECRB is parallel and extra-articular to the R-LCL. The ECRB is primarily a dynamic stabilizer of wrist extension and contributes to elbow stability in varus by exerting tension on the lateral epicondyle. A cadaveric study examined 30 elbows to define the anatomical origin of the ECRB tendon and its relationship to surgical landmarks. It found the ECRB originates beneath the distal supracondylar ridge, averaging 13 ± 2 mm in length and 7 ± 2 mm in width, and found it converges with the extensor digitorum communis distal to the radio-capitellar joint [23].

Other Cadaveric studies have elucidated the biomechanical significance of the whole Lateral Collateral Ligament–Capsule Complex (LCL-cc). One study analyzed the effects of staged release of the LCL-cc in 8 cadavers and found that loss of the anterior two-thirds or more of the LCL-cc significantly increases the overall mean contact pressure on the coronoid, especially the medial coronoid, under both gravity varus and weighted varus [24]. The authors concluded that the LCL-cc plays a role in the distribution of coronoid contact pressure against gravity varus loads.

In a biomechanical cadaveric study of ten elbows, sequential sectioning was used to quantify lateral elbow laxity through dynamic ultrasound measurement of joint gapping (Da#) at 60° of elbow flexion. Sectioning the Common Extensor Origin (CEO) alone led to an average increase of 8.5 ± 4.6% compared to the intact state.

Additional pie-crusting of the R-LCL caused a further increase of 6.9 ± 3.9%.

Finally, complete R-LCL release produced a cumulative increase of 25.7 ± 7.2%, nearly tripling the baseline value.

These results highlight the R-LCL’s critical role in lateral elbow stability and suggest that even an isolated strain can produce significant articular gapping even in the absence of a typical PLRI [17]. R-LCL elongation determines annular ligament incompetency as the two structures are intimately connected. When the annular ligament is placed anatomically under the radial neck at 90° of flexion, the condition of the so-called loose collar sign is reached. A lowered annular ligament does not work anymore as a restraint to the anterior migration of the radial head and anteroposterior radial head shift. In more advanced conditions, a lesion in the anterolateral capsule is visible as well.

These findings support the idea that horizontal microinstability—defined as non-rotational anterior–posterior gapping—is a legitimate and possibly underdiagnosed pattern in the elbow.

## 4. The SMILE Model and Horizontal Instability

**Figure 2:**
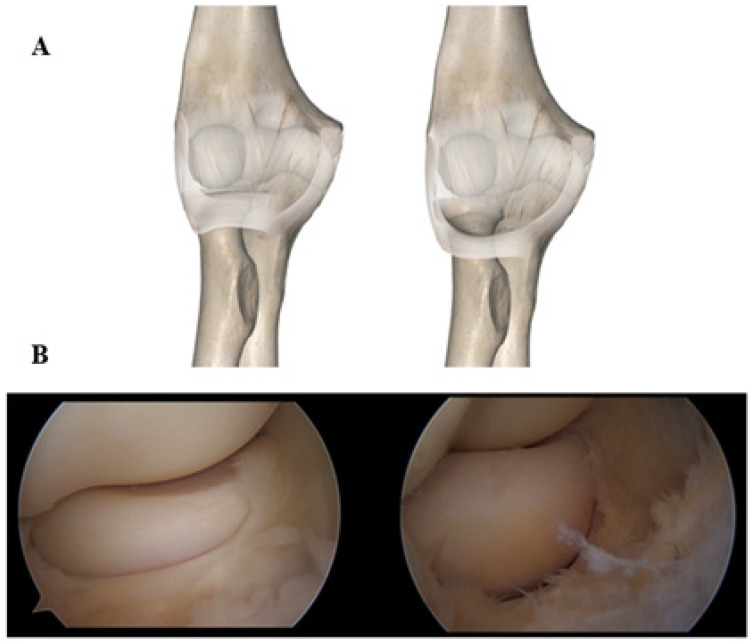
Graphical representation (**A**) and arthroscopic view (**B**) of the Loose Collar Sign (LCS) in controls (**left**) and in pathological patients (**right**).

While vertical instability in the elbow, defined as arthroscopically as the separation of ulna and humerus that happens during dislocation or rotatory laxity, has been extensively characterized, the concept of horizontal instability remains poorly defined in clinical orthopedics. In the SMILE model, particular emphasis is placed on the anterior portion of the radial head as a key pivot for detecting subtle joint incongruity [15,25]. Because of the incompetence of the Radial band and annular ligament, there is a natural tendency of the radial head to displace anteriorly. This anteroposterior shift in the radial head into the proximal radioulnar joint (PRUJ) can be described as horizontal instability in an arthroscopic setting. Arthroscopically, major intra-articular signs of SMILE laxity are the following:

- Annular Drive Through (ADT). This test tends to anteroposteriorly (horizontally) displace the radial head inside the annular ligament. It is performed with the arthroscope in the posterolateral portal, while the posterior radial head is pushed forward with the thumb. If the maneuver allows enough space for a 4.2 mm shaver to slide between the radial head and the annular ligament, with minimal resistance, then it is considered positive.
- Loose Collar Sign (LCS). Looking from the anteromedial portal, annular ligament laxity makes visible the radial neck below the cartilaginous portion of the head, with the elbow at 90° of flexion (Figure 2).
- Pull-Up Sign (PRS). This sign shows laxity of the radial component of the lateral collateral ligament; a positive test is achieved by pulling more than 1 cm of the R-LCL/annular ligament up towards the capitellum, with a grasper introduced from the anterolateral portal.

Other signs usually found on cases with horizontal instability/laxity of the R-LCL are as follows:

- Plica in the posterolateral compartment.
- Synovitis anterior to the radial head, or anteromedially at the insertion of the annular ligament to the sigmoid notch.
- A local “Chondropathy of the Lateral Aspect of the Capitellum” (CLAC lesion).
- A tear of the capsule of the radiocapitellar joint.
- Chondral lesions/chondropathy of the anterosuperior aspect of the radial head, where it engages the sigmoid notch, with the forearm in pronation
- Chondropathy of the lateral facet of the trochlear ridge is very likely a sign of a radial head that overpressures in varus and flexion.

Yet despite mounting evidence, horizontal instability remains absent from most elbow instability classifications, potentially delaying appropriate diagnosis and treatment in patients with persistent lateral pain. Horizontal instability can be defined as the main direction of instability and it is confirmed if vertical instability is minimal or absent. In the case of a global “opening” of the joint, while stressed, a condition of global laxity has to be considered. Global laxity of the elbow and lateral elbow pain is a very understudied field. When both medial and lateral elbow complexes have been involved in an injury, and initial treatment is delayed or does not provide a stable joint, instability after reduction may persist [5]. In such scenarios, medial and lateral elbow pain are expected, and simple repair of the ligaments may not suffice due to injury chronicity [26]. Several reconstruction techniques exist, attempting to address both elbow sides with a single or a double loop of the harvested tendon around the humerus and ulna [27,28,29,30]. One of the largest cohorts from these studies presented the outcomes of 14 patients with residual medial and lateral elbow instability, eight of them with a history of previous operation because of trauma. The authors described a single box-loop technique using a palmaris longus allograft, which was passed through the humerus and ulna and sutured to itself. At final follow-up, 13/14 patients had improved functionality and the mean gain in elbow ROM was 32 degrees [28].

## 5. Diagnostic Pathway

Diagnosis of SMILE requires an integrative approach combining patient history, dynamic imaging, and intraoperative assessment. Traditional clinical tests, such as the pivot shift, are often negative in SMILE, necessitating alternative modalities.

Patients often complain of chronic lateral elbow pain that has been resistant to at least 6 months of physical therapy. Even though the Cozen test [31] will most likely be positive, additional maneuvers can reveal a possible minor instability of the elbow: SALT (Supination and AnteroLateral pain Test) and PEPPER (Posterior Elbow Pain by Palpation–Extension of the Radiocapitellar joint) [32]. These patients will complain not only of the common pain elicited by palpation of the lateral epicondyle, which is the insertion of the ERCB, but also of the anterior and posterior aspects of the elbow, highlighting the presence of intra-articular pathology. In contrast, patients suffering from atraumatic PLRI will show evidence of postero-lateral instability, such as the chair rise test, push-up test, posterolateral drawer test, pivot shift test, pivot shift apprehension test, or varus stress test [33].

Concerning first-level imaging, ultrasound has been shown to be extremely helpful. The HELP-US test is an ultrasonographic maneuver that places the elbow in a varus-flexion-weighted position. In a prospective ultrasound study of 65 elbows, the testing maneuver was performed with the elbow flexed at 70° and a 3 kg load suspended at the wrist. Among 35 patients with suspected minor instability, the mean lateral joint space widened by 26.7%, compared to just 3.2% in 30 healthy controls—a statistically significant difference (*p* < 0.01). These findings suggest that the HELP-US test is a reliable, non-invasive tool for detecting subtle lateral elbow laxity in patients with atraumatic pain and may help expedite advanced imaging in select cases [18]. Therefore, dynamic ultrasound also offers a non-invasive method to compare side-to-side differences in joint laxity.

When correlated with CT-arthrography, these techniques enhance diagnostic specificity. In a retrospective imaging study of 90 elbow CT arthrograms, the SMILE Index was developed; it is a semiquantitative scoring system ranging from 0 to 8, designed to evaluate features of minor lateral elbow instability. The score integrates findings such as chondromalacia, ligament laxity, synovial thickening, and joint asymmetry. The index demonstrated excellent intra-reader agreement (κ = 0.94, 87% concordance) and substantial inter-reader reliability (κ = 0.75, 67% concordance), confirming its reproducibility. With a median SMILE score of 4, the tool provides a reliable radiological adjunct to clinical diagnosis in patients with lateral elbow pain (Table 1) [16].

Symptom duration has been shown to correlate with the likelihood of intra-articular pathology in patients presenting with lateral elbow pain. In an arthroscopic study, 85.7% of patients with symptoms of atraumatic lateral elbow pain persisting beyond six months demonstrated intra-articular abnormalities, and 48.6% showed arthroscopic signs of patholaxity of the radial band of the lateral collateral ligament, despite no history of trauma or dislocation [15]. Similarly, another study found that 59% of patients undergoing arthroscopy for chronic lateral elbow pain had cartilage degeneration in the lateral compartment, findings often missed on imaging and attributed to epicondylitis [34]. These results suggest that a symptom duration longer than six months significantly increases the probability of underlying structural lesions, supporting its role as a key factor in risk stratification and guiding advanced imaging or surgical referral.

## 6. Surgical Controversies: The Role of Lateral Release

The Nirschl procedure, a widely adopted surgical treatment for lateral epicondylitis, involves releasing the origin of the ECRB. While it has demonstrated success in relieving symptoms of chronic tendinopathy, mainly pain, its implications for elbow stability remain insufficiently studied. Nirschl originally advocated for this release to address degenerative changes in the ECRB origin and eliminate pain caused by repetitive microtrauma [35].

However, concerns have emerged about whether releasing the ECRB origin—particularly when combined with partial R-LCL detachment—might predispose patients to lateral elbow laxity or SMILE. Indeed, in cadaveric and ultrasound studies, ECRB detachment worsened joint laxity in varus stress [17,36].

This has prompted debate on whether the traditional Nirschl release may contribute to secondary instability in patients with unrecognized R-LCL pathology. In a recent ultrasonographic study, lateral elbow instability was evaluated by placing the joint under varus and axial load during ultrasound examination. The authors found that patients with chronic lateral pain and suspected SMILE exhibited increased radio-capitellar translation under dynamic stress [18].

Despite the widespread use of lateral release, no prospective controlled trials have evaluated its biomechanical consequences on elbow stability in vivo. A recent retrospective cohort study, which included a total of 62 patients suffering from lateral epicondylitis, compared the results of the arthroscopic Nirschl procedure versus arthroscopic CEO suture anchor repair. At a minimum 1-year follow-up, the postoperative MEPS and QuickDASH scores were better in the repair group than in the debridement alone group [37]. Regardless, the absence of high-level evidence represents a critical gap in current surgical practice, particularly in patients presenting with chronic lateral elbow pain, where subtle instability might be a contributing factor.

## 7. Arthroscopic Stabilization Techniques

Arthroscopic approaches to lateral elbow instability have evolved as minimally invasive alternatives to open ligament reconstruction. In SMILE, stabilization focuses on restoring tension in the lateral ligamentous and capsular structures without over-constraining motion.

**Figure 3:**
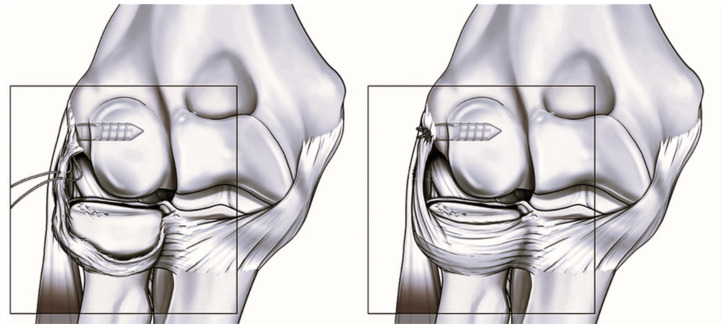
R-LCL applications in SMILE patients.

In a retrospective case series of 27 patients with chronic lateral elbow pain unresponsive to conservative treatment, the authors performed arthroscopic plication of the radial band of the lateral collateral ligament (R-LCL) [25]. All patients showed intra-articular abnormalities and signs of ligamentous patholaxity but had no history of trauma or frank instability. At a median 2-year follow-up, the Single Assessment Numeric Evaluation (SANE) score improved from 30 to 90, and 96.3% of patients reported good or excellent subjective outcomes. Postoperative QuickDASH was 9.1, and the Oxford Elbow Score was 42, indicating substantial functional recovery. However, 41% of patients experienced some restriction in elbow range of motion, with a minority not achieving full extension or flexion. These findings suggest that arthroscopic R-LCL plication is effective in improving pain and function in SMILE patients, though it may carry a risk of mild postoperative stiffness (Figure 3) [25].

In a cohort of 20 patients with grade 1 or 2 PLRI, the authors evaluated a modified arthroscopic lateral collateral ligament (LCL) imbrication technique. Mean Mayo Elbow Performance Score increased from 48 to 88.9 (*p* < 0.001), and QuickDASH improved from 53 to 10.3 (*p* < 0.001) at a minimum 2-year follow-up. Eighteen of 20 patients were objectively stable postoperatively. Minor complications included stiffness (n = 1) and knot tenderness (n = 2) [38].

Another study evaluated eight patients diagnosed with atraumatic PLRI who underwent arthroscopic LCL (lateral collateral ligament) imbrication [33]. At a mean 16-month follow-up, QuickDASH scores improved from 55.6 to 7.6 (*p* = 0.004), MEPI scores from 50 to 96.9 (*p* = 0.000), and VAS pain scores from 8.1 to 1.3 (*p* = 0.000). This study supports the clinical value of arthroscopic imbrication in restoring stability and relieving pain in atraumatic PLRI, even when performed alongside treatment of associated pathology such as epicondylitis or plica.

To address minor instability of the lateral elbow in a minimally invasive manner, a technique article described a modified arthroscopic LCL imbrication technique involving a doubled suture passed through the lateral capsule, LCL complex, and anconeus, secured using a Nice knot. This approach aims to restore elbow stability by reinforcing the posterolateral structures without extensive dissection or grafting, offering an anatomically respectful alternative to open reconstruction [39].

This technique was subsequently applied in a prospective clinical study of 43 patients with grade I–II posterolateral rotatory instability (PLRI). The authors observed significant functional improvement, with the Mayo Elbow Performance Score increasing from a median of 45 preoperatively to 95 at 12-month follow-up (*p* < 0.001). Full range of motion was restored in over 95% of patients, with only mild and transient complications such as knot discomfort in the early cohort. These results confirmed that arthroscopic LCL imbrication is an effective and well-tolerated intervention for low-grade PLRI [40].

Nevertheless, arthroscopic stabilization is not without risks. Loss of terminal extension has been reported postoperatively in both open and arthroscopic approaches. Careful patient selection and early mobilization protocols are essential to mitigate complications.

## 8. Proposed Clinical Algorithm

**Figure 4:**
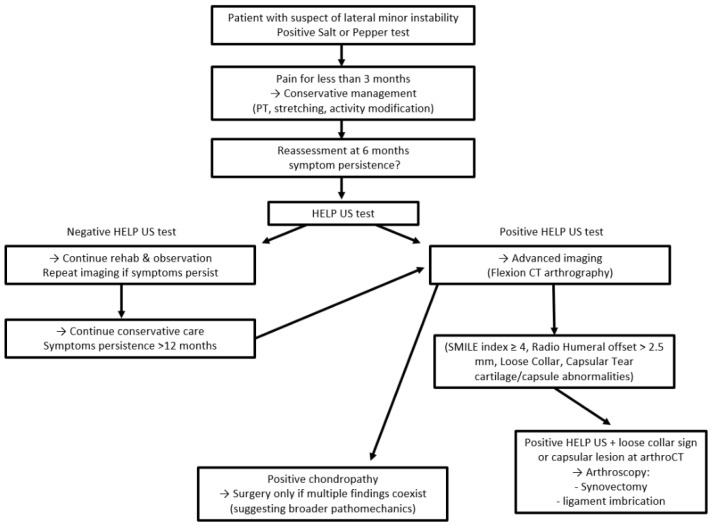
Proposed clinical algorithm to guide the diagnostic and therapeutic management of lateral elbow pain with suspected microinstability.

Based on emerging clinical evidence and imaging validation, a structured algorithm is proposed to guide the diagnostic and therapeutic management of lateral elbow pain with suspected microinstability (Figure 4). The following decision-making tree integrates symptom duration, dynamic ultrasound findings, and advanced imaging criteria to stratify patients and identify those likely to benefit from surgical intervention.

In patients presenting with lateral elbow pain for less than three months, conservative management, including physical therapy, exercises, activity modification, and anti-inflammatory strategies, should be pursued. No immediate imaging is required at this stage.

At the six-month mark, patients who remain symptomatic should undergo clinical reassessment. If the HELP-US test is positive—indicating lateral joint gapping under varus load—advanced imaging is warranted, preferably flexion CT arthrography. A negative HELP-US test justifies continued observation and rehabilitation, with repeat imaging if symptoms persist.

If imaging confirms findings consistent with microinstability—such as a SMILE index score of 4 or more, a radial head offset > 2.5 mm, or cartilage and capsule abnormalities—then surgical consultation is recommended. 

Within this cohort, patients with both positive HELP-US and true loose collar sign or capsular lesion at arthroCT are candidates for arthroscopic intervention, including synovectomy, plica debridement, and radial band or annular ligament imbrication.

In cases of isolated chondropathies, surgical intervention should only be considered if multiple positive findings coexist, suggesting a broader pathomechanical process.

This algorithm aims to reduce the rate of diagnostic uncertainty, avoid unnecessary procedures, and identify patients most likely to benefit from stabilization surgery. Prospective validation across diverse populations remains an ongoing need.

## Figures and Tables

**Table 1:** Variables for assessing elbow instability using the SMILE index. SMILE = Symptomatic Minor Instability of The Lateral Elbow; ^†^ = Percentage of exposure of the radial head side.

	0	1	2
**Radial head side chondromalacia**	Absent	Present	
**Lateral humeral capitellum chondromalacia**	Absent	Present	
**Humeral trochlear ridge chondromalacia**	Absent	Present	
**Annular ligament laxity**	Absent	≤50% ^†^	>50% ^†^
**Synovial thickening**	Absent	Present	
**Humeroradial joint asymmetry**	Absent	Present	
**Capsular tear**	Absent	Present	

## Abbreviations

R-LCL	Radial-Lateral Collateral Ligament
SMILE	Symptomatic Minor Instability of the Lateral Elbow
PLRI	Posterolateral Rotatory Instability
ECRB	Extensor Carpi Radialis Brevis
CT	Computed Tomography
US	UltraSonographic
MUCL	Medial Ulnar Collateral Ligament
MRI	Magnetic Resonance Imaging
LCLC	Lateral Collateral Ligament Complex
LUCL	Lateral Ulnar Collateral Ligament
LCL-cc	Lateral Collateral Ligament–Capsule Complex
CEO	Common Extensor Origin
PRUJ	Proximal RadioUlnar Joint
ADT	Annular Drive Through
LCS	Loose Collar Sign
PRS	Pull-Up Sign
CLAC	Chondropathy of the Lateral Aspect of the Capitellum
SALT	Supination and AnteroLateral pain Test
PEPPER	Posterior Elbow Pain by Palpation–Extension the Radiocapitellar Joint
LCL	lateral Collateral Ligament

## References

[1] Shiri R., Viikari-Juntura E. (2011). Lateral and medial epicondylitis: Role of occupational factors. Best Pract. Res. Clin. Rheumatol..

[2] Herquelot E., Bodin J., Roquelaure Y., Ha C., Leclerc A., Goldberg M., Zins M., Descatha A. (2013). Work-related risk factors for lateral epicondylitis and other cause of elbow pain in the working population. Am. J. Ind. Med..

[3] Walz D.M., Newman J.S., Konin G.P., Ross G. (2010). Epicondylitis: Pathogenesis, imaging, and treatment. Radiographics.

[4] Johns N., Shridhar V. (2020). Lateral epicondylitis: Current concepts. Aust. J. Gen. Pract..

[5] Marinelli A., Guerra E., Rotini R. (2016). Elbow instability: Are we able to classify it? Review of the literature and proposal of an all-inclusive classification system. Musculoskelet. Surg..

[6] O’Driscoll S.W., Bell D.F., Morrey B.F. (1991). Posterolateral rotatory instability of the elbow. J. Bone Jt. Surg..

[7] O’Driscoll S.W. (2000). Classification and evaluation of recurrent instability of the elbow. Clin. Orthop. Relat. Res..

[8] Chanlalit C., Limsricharoen W. (2013). Posterolateral rotatory instability from multiple steroids injections for tennis elbow: A case report. J. Med. Assoc. Thai..

[9] Chanlalit C., Phorkhar T. (2015). Posterolateral Rotatory Apprehension Test in Tennis Elbow. J. Med. Assoc. Thai..

[10] Murray I.R., Goudie E.B., Petrigliano F.A., Robinson C.M. (2013). Functional anatomy and biomechanics of shoulder stability in the athlete. Clin. Sports Med..

[11] Housset V., Ho S.W.L., Lädermann A., Phua S.K.A., Hui S.J., Nourissat G. (2024). Multidirectional instability of the shoulder: A systematic review with a novel classification. EFORT Open Rev..

[12] Kani K.K., Mulcahy H., Chew F.S. (2016). Understanding carpal instability: A radiographic perspective. Skelet. Radiol..

[13] Beutel B.G., Konstanty J., Beeker R.W., Rehman U.H. (2025). Carpal Ligament Instability.

[14] Taleisnik J. (1988). Current concepts review. Carpal instability. J. Bone Jt. Surg..

[15] Arrigoni P., Cucchi D., D’aMbrosi R., Butt U., Safran M.R., Denard P., Randelli P. (2017). Intra-articular findings in symptomatic minor instability of the lateral elbow (SMILE). Knee Surg. Sports Traumatol. Arthrosc..

[16] Zagarella A., Folco G., Monti C.B., Rizzo A., Arrigoni P., Vismara V., Cassin S., Gallazzi M.B. (2023). Semiquantitative index of symptomatic minor instability of the lateral elbow at CT arthrography (SMILE index): Clinical applicability and reproducibility study. Eur. Radiol..

[17] Arrigoni P., Cucchi D., Luceri F., Menon A., Zaolino C., Zagarella A., Catapano M., Radici M., Migliaccio N., Polli D. (2021). Lateral Elbow Laxity Is Affected by the Integrity of the Radial Band of the Lateral Collateral Ligament Complex: A Cadaveric Model with Sequential Releases and Varus Stress Simulating Everyday Activities. Am. J. Sports Med..

[18] Traverso A., Vismara V., Cassin S., Zagarella A., Randelli P., Arrigoni P. (2025). Ultrasonography of lateral elbow pain through a weighted varus flexion position contributes to detect minor instabilities. JSES Int..

[19] Morrey B.F. (1996). Acute and Chronic Instability of the Elbow. J. Am. Acad. Orthop. Surg..

[20] Ring D., Jupiter J.B. (1998). Fracture-dislocation of the elbow. J. Bone Jt. Surg..

[21] Willemot L., Hendrikx F.R., Byrne A.-M., van Riet R.P. (2018). Valgus instability of the elbow: Acute and chronic form. Obere Extrem..

[22] Meyer M.A., Leversedge F.J., Catalano LW3rd Lauder A. (2024). Complex Elbow Fracture-Dislocations: An Algorithmic Approach to Treatment. J. Am. Acad. Orthop. Surg..

[23] Cohen M.S., Romeo Aa Hennigan S.P., Gordon M. (2008). Lateral epicondylitis: Anatomic relationships of the extensor tendon origins and implications for arthroscopic treatment. J. Shoulder Elb. Surg..

[24] Kwak J.-M., Rotman D., Lievano J.R., Fitzsimmons J.S., O’Driscoll S.W. (2023). The role of the lateral collateral ligament-capsule complex of the elbow under gravity varus. J. Shoulder Elb. Surg..

[25] Arrigoni P., Cucchi D., D’Ambrosi R., Menon A., Aliprandi A., Randelli P. (2017). Arthroscopic R-LCL plication for symptomatic minor instability of the lateral elbow (SMILE). Knee Surg. Sports Traumatol. Arthrosc..

[26] Hackl M., Heinze N., Wegmann K., Lappen S., Leschinger T., Burkhart K.J., Scaal M., Müller L.P. (2016). The circumferential graft technique for treatment of multidirectional elbow instability: A comparative biomechanical evaluation. J. Shoulder Elb. Surg..

[27] Aminata I.W., Efar T.S., Canintika A.F. (2019). Chronically unreduced elbow dislocation treated with box-loop ligament reconstruction: The first case series. J. Clin. Orthop. Trauma.

[28] Finkbone P.R., O’Driscoll S.W. (2015). Box-loop ligament reconstruction of the elbow for medial and lateral instability. J. Shoulder Elb. Surg..

[29] van Riet R.P., Bain G.I., Baird R., Lim Y.W. (2006). Simultaneous reconstruction of medial and lateral elbow ligaments for instability using a circumferential graft. Tech. Hand Up. Extrem. Surg..

[30] Sachinis N.P., Vasiadis I., Yiannakopoulos C.K., Givissis P. (2022). Modified Graft Loop Technique Augmented with Nonabsorbable Suture Tape for Chronic Elbow Dislocation. Tech. Hand Up. Extrem. Surg..

[31] Zwerus E.L., Somford M.P., Maissan F., Heisen J., Eygendaal D., van den Bekerom M.P. (2018). Physical examination of the elbow, what is the evidence? A systematic literature review. Br. J. Sports Med..

[32] Arrigoni P., Cucchi D., Menon A., Randelli P. (2017). It’s time to change perspective! New diagnostic tools for lateral elbow pain. Musculoskelet. Surg..

[33] Chanlalit C., Mahasupachai N., Sakdapanichkul C. (2022). Arthroscopic lateral collateral ligament imbrication for treatment of atraumatic posterolateral rotatory instability. J. Orthop. Surg..

[34] Rajeev A., Pooley J. (2009). Lateral compartment cartilage changes and lateral elbow pain. Acta Orthop. Belg..

[35] Gunn C.C. (1980). Tennis elbow. The surgical treatment of lateral epicondylitis. J. Bone Jt. Surg..

[36] Arrigoni P., Cucchi D., Luceri F., Zagarella A., Catapano M., Menon A., Bruno V., Gallazzi M., Randelli P.S. (2021). Ultrasound evaluation shows increase in laxity after partial common extensor origin detachment but not after additional lesion of the radial band of the lateral collateral ligament. Knee Surg. Sports Traumatol. Arthrosc..

[37] Yao L., Xiong Y., Ma W., Li J., Tang X. (2025). Arthroscopic Suture Anchor Repair and Debridement for Refractory Lateral Epicondylitis Show Significant Clinical Improvement. Arthrosc. J. Arthrosc. Relat. Surg..

[38] Kohlprath R., Vuylsteke K., van Riet R. (2022). Arthroscopic lateral collateral ligament imbrication of the elbow: Short-term clinical results. J. Shoulder Elb. Surg..

[39] Sachinis N.P., Yiannakopoulos C.K., Beitzel K., Koukos C. (2023). Arthroscopic Modified Elbow Lateral Collateral Ligament Imbrication: An Operative Technique. Arthrosc. Tech..

[40] Koukos C., Sachinis N.P., Sidiropoulos K., Kotsapas M., Bilsel K., Montoya F. (2025). Arthroscopic lateral collateral ligament imbrication for the treatment of posterolateral rotatory elbow instability. JSES Int..

